# The effect of steroid pretreatment of deceased organ donors on liver allograft function: A blinded randomized placebo-controlled trial

**DOI:** 10.1016/j.jhep.2012.01.020

**Published:** 2012-06

**Authors:** Stefan Amatschek, Julia Wilflingseder, Mario Pones, Alexander Kainz, Martin Bodingbauer, Ferdinand Mühlbacher, Robert M. Langer, Zsuzsanna Gerlei, Rainer Oberbauer

**Affiliations:** 1 KH Elisabethinen, Linz, Austria; 2 Department of Internal Medicine 3, Medical University of Vienna, Austria; 3 Department of Transplantation, Medical University of Vienna, Austria; 4 Department of Transplantation and Surgery, Semmelweis University, Budapest, Hungary

**Keywords:** BCAR, biopsy-confirmed rejection, ALT, alanine transaminase, AST, aspartate transaminase, GGT, glutamyl transpeptidase, AP, alkaline phosphatase, RAI, reaction activity index, BIL, total bilirubin, ALB, serum albumin, Liver transplantation, Steroid, Donor pretreatment, Early liver allograft function

## Abstract

**Background & Aims:**

Brain death-associated inflammatory response contributes to increased risk of impaired early liver allograft function, which might be counterbalanced by steroid pretreatment of the organ donor. The aim of this randomized controlled trial was to elucidate whether steroid pretreatment of liver donors improves early liver allograft function, prevents rejection and prolongs survival.

**Methods:**

A placebo-controlled blinded randomized clinical trial was performed in three different centers in Austria and Hungary between 2006 and 2008. Ninety deceased organ donors received either 1000 mg of methylprednisolone or placebo 6 h before recovery of organs. The primary end point was the concentration slope of transaminases within the first week. The secondary end point included survival and biopsy-confirmed acute rejection (BCAR) within 3 years after transplantation.

**Results:**

Of the 90 randomized donors, 83 recipients were eligible for study. The trajectories of ALT and AST were not different between treatments (*p* = 0.40 and *p* = 0.13, respectively). Eight subjects died in the steroid and 13 in the placebo group within 3 years after engraftment (RR = 0.63 95% CI [0.29, 1.36], *p* = 0.31). Eleven recipients experienced biopsy-confirmed rejection (BCAR) in the steroid and 11 in the placebo group (RR = 1.02 95% CI [0.50, 2.10], *p* = 1.00). No effect modification could be identified in the predefined strata of donor age, sex, cold ischemic time, and cause of donor death.

**Conclusions:**

Steroid pretreatment of organ donors did not improve outcomes after liver transplantation.

## Introduction

Liver transplantation is the only available treatment of end stage liver disease. However, a raising demand for liver transplant organs has increased the median waiting time for liver allografts in the Eurotransplant region from 4 months in 2000 to 14 months in 2009 [1]. Even worse, approximately 20% of all patients on the waiting list for liver transplantation died in 2009.

Thus donor criteria have been liberalized in the last decade as a consequence of the imbalance between supply and demand. For example, the median age of liver donors increased from 26 to 52 years between 1990 and 2009. While donor age has been considered a major risk factor for long time graft survival [2,3], there is an increasing evidence that also consequences of donor brain death in the graft might exert adverse effects on transplant recipients.

It has been shown in kidney transplantation that graft survival from living donors is significantly longer when compared to deceased donor allografts [4,5]. Furthermore, brain death triggers a complex series of pathophysiological changes that also drive alterations of gene expression in transplant organs [6–10], although donor organs derived from living or deceased donors cannot be distinguished on a morphological basis. We previously showed in kidney grafts that gene expression profiles derived from deceased organs are characterized by a severe pro-inflammatory state which is not observed in organs from living donors [11].

Recently, we elucidated that pretreatment of deceased organ donors with corticosteroids led to a significant reduction of the molecular inflammation signature in transplant kidneys even though the rate of delayed graft function remained unchanged [12].

The influence of steroid pretreatment on the outcome after liver transplantation has been evaluated in a previous study [13]. In this publication, the authors claimed that steroid donor treatment significantly ameliorated ischemia reperfusion injury determined by AST levels on day one after transplantation. However, the effect of steroid pretreatment on the genome wide inflammatory signature as well as the trajectories of transaminases and hard long-term outcomes remained elusive.

Thus, the goal of the present study was to test in a blinded, randomized, placebo-controlled clinical trial whether steroid pretreatment of the deceased organ donor caused a reduced release of transaminase from the donor liver and subsequently less rejection and longer survival.

## Materials and methods

### Study design

The liver grafts recovered from multiorgan donors of our recently published randomized, blinded, placebo-controlled trial were evaluated [12]. The trial is registered under controlled-trials.com registration number ISRCTN78828338. Out of the total eligible donors (n = 269), 90 liver donors (39 from Vienna, 39 from Budapest and 5 from Linz) were randomized equally in the steroid or placebo group. The detailed CONSORT criteria can be found in Kainz *et al.* [12]. Briefly, after brain death was declared, deceased donors received either a single injection of 1000 mg methylprednisolone or placebo (0.9% saline) between 6 and 3 h before organ recovery. All organs were perfused with a histidine-ketoglutarate preservation solution during organ procurement. Liver transplant recipients from the transplant centers in Vienna (Austria) and Budapest (Hungary) were included in our trial. The donor demographics were collected at the study website http://www.meduniwien.ac.at/nephrogene/trials/ and the liver recipient demographics and follow-up data of the recipient were collected by the local transplant coordinators.

Donors were equally randomized to steroid or placebo treatment based on a permuted block design with block sizes of four (https://www.meduniwien.ac.at/randomizer). Central randomization was stratified for donor age above 50 years through our study website and concealed until data analysis. The randomization order did not have a repeating sequence and the randomization code was not revealed to recipients or investigators. The local transplant coordinators, enrolled donors, recipients and investigators were blinded for the allograft treatment.

The study protocol was approved by the institutional review board (Ethical Committee of the Medical University of Vienna, Vienna, Austria [study protocol EK-067/2005; to be found at http://ohrp.cit.nih.gov/search]), TUKEB-Hungary and Eurotransplant (study protocol 6021KAC06) at each study site.

### Outcomes and sample size calculation

Our primary end point was the serum level trajectories of transaminases (alanine transaminase (ALT) and aspartate transaminase (AST)) within the first week after engraftment.

We calculated a detectable difference in the slopes of 0.65 between the groups at a standard deviation of 0.3, a correlation coefficient of 0.7 in the sequential individual measurements and the assumption that a slope of the log transformed AST levels in the first week is 0.5. Study power was 80% at an alpha value of 0.05 assuming a 10% drop-out rate to detect such difference. Exclusion criteria were fulminant liver failure, multiorgan transplantation or retransplantation and HIV-positive recipients.

Liver specific data, which were recorded daily in the first week of the trial, included gamma glutamyl transpeptidase (GGT), alkaline phosphatase (AP), albumin as well as total bilirubin.

The secondary end point included graft loss, mortality and BCAR (Rejection Activity Index (RAI) score ⩾3) within 3 years after transplantation.

Effect modification of the predefined subgroups donor age, sex, cold ischemic time, and cause of donor death were evaluated by interaction analyses.

### Statistical analysis

Demographic baseline characteristics were compared using *t* tests for continuous data and Chi-square tests or Fisher exact tests for categorical data. Trajectories of various liver parameters after transplantation were analyzed using a linear mixed model with time, therapy as well as the transplantation center as independent variables. Kaplan–Meier plots were used to visualize time to acute rejection episodes and the combined graft loss/death. Differences between groups were calculated by the log-rank test. Data are provided as mean ± SD if not otherwise indicated. *p* values <0.05 were considered to be statistically significant. All analyses were performed with SAS for Windows 9.2 (Cary, NJ, USA).

## Results

### Baseline characteristics of organ donors and recipients

**Fig. 1:**
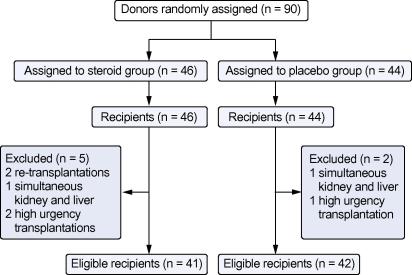
**CONSORT flowchart of organ donor and liver graft recipients**.

Of the 90 randomized donors, seven allografts were allocated to recipients with study exclusion criteria (2 retransplant, 2 simultaneous kidney and liver, 3 high urgency transplantations). Of the remaining 83 donor organs, 41 organs were procured from steroid treated and 42 from placebo treated donors between 2006 and 2008 (see CONSORT flowchart in Fig. 1). Age, sex and clinical indices of donors and recipients are provided in Table 1. Mean donor age and recipient age were not different in both groups (47 ± 12 and 48 ± 13 years in the placebo group *vs.* 46 ± 12 and 49 ± 9 years in the treatment group). Accordingly, mean MELD score of the recipient and cold ischemic time did not reveal significant differences between the study arms (16 ± 6 and 440 ± 123 min in the control group *vs.* 15 ± 6 and 444 ± 136 min in the steroid group).

The immunosuppressive regime of all subjects included 40 mg dexamethasone intraoperatively, 3 days of ATG induction (Thymoglobuline®, Genzyme 2.5 mg/kg/d) and CNI maintenance therapy. Rejections were treated with 3 days of 500 mg prednisolone.

### Primary and secondary study end points

**Fig. 2:**
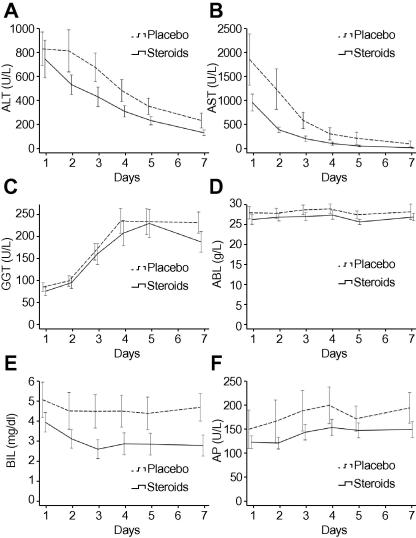
**Trajectories of liver function parameters in the first week after transplantation**. Mean values and standard errors of mean from day 1 to day 7 after transplantation are shown for (A) alanine transaminase (ALT) (*p* = 0.40), (B) aspartate transaminase (AST) (*p* = 0.13), (C) glutamyl transpeptidase (GGT) (*p* = 0.75), (D) serum albumin (ALB) (*p* = 0.32), (E) total bilirubin (BIL) (*p* = 0.14) and (F) alkaline phosphatase (AP) (*p* = 0.30).

Biochemical indicators of liver cell necrosis over the first week are depicted in Fig. 2. Trajectories of alanine and aspartate transaminases (ALT and AST) were not different between the two treatment groups (*p* = 0.40 and *p* = 0.13, respectively). Mean ALT levels decreased between day 1 and day 7 post-transplantation from 784 ± 1025 U/L to 156 ± 148 U/L in the steroid group while levels decreased from 870 ± 904 U/L to 261 ± 402 U/L in the placebo group. Similarly, mean AST levels dropped from 1002 ± 1130 U/L to 59 ± 58 U/L in the steroid group compared to 1898 ± 3364 U/L to 140 ± 398 U/L in the placebo group.

Accordingly, the trajectories of glutamyl transpeptidase (GGT, *p* = 0.75), total bilirubin (BIL, *p* = 0.14), serum albumin (ALB, *p* = 0.32) and alkaline phosphatase (AP, *p* = 0.30) within 7 days after transplantation were not different between the two groups.

The relative risk of mortality in the steroid compared to the placebo group was 0.63 (95% CI [0.29, 1.36], *p* = 0.31). The relative risk for BCAR in the steroid compared to the placebo arm was 1.02 (95% CI [0.50, 2.10], *p* = 1.00).

**Fig. 3:**
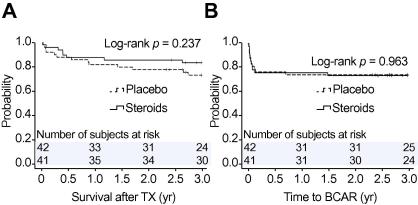
**Kaplan–Meier estimates of (A) survival (*p*** **=** **0.24) and (B) biopsy-confirmed rejection (log-rank *p*** **=** **0.96) risks are shown for steroid pretreated livers and placebo**. The number of subjects at risk is provided above the x-axis. Given *p* values were derived from log-rank tests. BCAR, biopsy-confirmed rejection; TX, transplantation.

The probability of survival and BCAR episodes over a follow-up period of 3 years was not statistically different in the two treatment arms (Fig. 3A and B). In the first year, 6 patients (15%) experienced graft loss in the steroid group *vs.* 10 patients (24%) in the placebo group (*p* = 0.41). One graft loss in the placebo group did not lead to death because of high urgency retransplantation. Acute rejection within the first 3 months occurred in 10 recipients (24%) in the steroid group *vs.* 10 patients (24%) in the placebo group (*p* = 1.00).

**Fig. 4:**
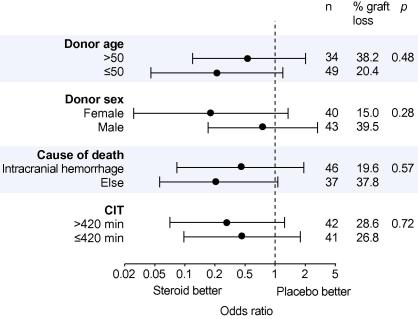
**Graft loss by various donor characteristics**. Indicated *p* values represent the interaction between treatment and characteristics.

We did not find any effect modification for the variables donor age, sex, cold ischemic time, and cause of donor death (Fig. 4).

## Discussion

Our study showed that systemic administration of 1000 mg methylprednisolone to the deceased organ donor did not significantly ameliorate liver allograft function or mortality or rejection within the first weeks after engraftment.

This result is in line with our observation that steroid pretreatment did not reduce the incidence or duration of acute renal transplant failure in kidney allograft recipients [12]. On the other hand, this finding contradicts a similar trial that has claimed a protective positive effect of steroid treatment in deceased donor liver transplantation [13,14]. It is therefore essential to try to explain these distinct results of both studies.

First, it seems necessary to clarify whether steroid administration was effective in terms of suppression of inflammation in the donor organ. To address this question, we have previously shown in kidney transplants, using genome wide expression profiling, that steroid treatment results in a pronounced suppression of inflammation/immune response-associated genes [12,15–17]. These data suggest that dose and timing of the blinded intervention were chosen correctly and randomization worked fine.

Furthermore, different experimental parameters between both trials could eventually explain varying outcomes. Administration of the corticosteroid was somewhat different since, in our study, donors received one single shot of 1000 mg whereas Kotsch *et al.* applied 250 mg initially followed by continuous infusion of 100 mg/h. In addition, the primary study end points were transaminases levels on day one post-transplantation in the trial of Kotsch *et al.* while the present study evaluated the trajectories of transaminases as a more stable measurement of a treatment effect. Another difference was the number of study sites, which has an effect on the balance of internal and external validity, i.e. generalizability of findings. Both studies found no significant differences between rejection rates [13].

Our study, however, was not powered to detect a statistical difference of transaminase trajectories between groups below 0.65. We considered a smaller difference in transaminase trajectories not to be of sound clinical significance. Donor organ quality determined by donor age, which predicts early and long term outcome [18], was not different between groups. A definitive strength of our study is the evaluation of the steroid intervention on hard study end points such as mortality and biopsy-confirmed rejection over a 3-year period.

In summary, liver ischemia reperfusion injury is obviously by far more complex than expected, so that a reductionist view limiting interventions to steroid pretreatment does not seem to be successful. Thus, although the brain death-related immune response might play an essential role during ischemia reperfusion injury and subsequent transplant dysfunction, it seems that this immune response itself is not a causative factor but that other factors must exist. Further efforts are therefore required in order to significantly improve ischemia reperfusion injury in the context of liver transplantation.

## Conflict of interest

The authors who have taken part in this study declared that they do not have anything to disclose regarding funding or conflict of interest with respect to this manuscript.

## Trial registration

Controlled-trials.com number ISRCTN78828338.

## Figures and Tables

**Table 1:** **Demographic data of donors and recipients stratified by treatment**.

^a^*p* = 0.04 (Fisher’s exact test).
